# Validity and Reliability of Wearable Technology Devices during Simulated Pickleball Game Play

**DOI:** 10.3390/sports12090234

**Published:** 2024-08-28

**Authors:** James W. Navalta, Bryson Carrier, Matahn Blank, Setareh Zarei, Dustin W. Davis, Micah Craig, Olivia R. Perez, Jacob Baca, Thea S. Sweder, Tashari Carballo, Jamaal Bovell

**Affiliations:** Department of Kinesiology and Nutrition Sciences, University of Nevada, Las Vegas, NV 89154, USA

**Keywords:** accuracy, consistency, paddle sport, Garmin Instinct, Polar Vantage M2

## Abstract

Pickleball is a popular sport. Also popular is wearable technology usage. Because the validity and reliability of wearable technology during pickleball is unknown, the purpose of this research was to evaluate the ability of common devices to return heart rate and estimated energy expenditure during pickleball activity. Twenty adult participants were outfitted with a portable metabolic unit and heart rate monitor (criterion measures). Experimental devices were a Garmin Instinct, Polar Vantage M2, Polar OH1, and Polar Verity Sense. Participants played simulated pickleball for 10 min. Validity measures included mean absolute percent error (MAPE) and Lin’s Concordance Correlation Coefficient (CCC), whereas reliability measures included coefficient of variation (CV) and intraclass correlation coefficient (ICC). The heart rate returned lower than 10% MAPE across all devices (Instinct = 5.73–6.32%, Verity Sense = 2.92–2.97%, OH1 = 3.39–3.45%) and greater than 0.85 CCC (Instinct = 0.85–0.88, Verity Sense = 0.96–0.96, OH1 = 0.93–0.94). The CV was below 10% (Instinct = 9.30%, Verity Sense = 2.68%, OH1 = 5.01%), and ICC was above 0.7 (Instinct = 0.77, Verity Sense = 0.98, OH1 = 0.91). The energy expenditure MAPE was greater than 10% (Instinct = 27.67–28.08%, Vantage M2 = 18.87–23.38%) with CCC lower than 0.7 (Instinct = 0.47–0.49, Vantage M2 = 0.62–0.63). Reliability thresholds were met in the Vantage M2 (CV = 6%, ICC = 0.98) but not in the Instinct (CV = 15%, ICC = 0.86). The Instinct was neither valid nor reliable for estimated energy expenditure, while the Polar Vantage M2 was reliable but not valid. All devices returned valid and reliable heart rates during pickleball.

## 1. Introduction

Pickleball is a racquet sport that has become the fastest growing sport in the United States [1]. While pickleball was first invented in 1965 [2], the number of players has rapidly increased in recent years, increasing from 4.8 million to 8.9 million from 2021 to 2022 alone [1]. Similar to tennis, it can be played as either singles or doubles [3], and involves players hitting a ball over a net on a rectangular court. Pickleball specifically incorporates paddles, a perforated ball, and a 6.1 × 13.4 m (20 ft × 44 ft) court [3]. Pickleball distinguishes itself from other racquet sports by requiring players to serve underhand as opposed to overhand [4]. To win a game, players must score 11 points with at least a 2-point lead over their opponents [3]. Pickleball is further set apart from other sports due to its inclusive nature, catering to diverse audiences from experienced athletes to older adults. Over half of pickleball players are 55 or older [2]. Accompanying pickleball’s recent increase in popularity is the rise of wearable technology, which the American College of Sports Medicine has identified as the world’s most prominent fitness trend of 2023 [5]. Wearable technology is defined as any compact device worn on the body that presents information and enables user interaction, whether through voice command or physical input [6]. Wearable devices can provide valuable information about physiological measures during physical activity, such as heart rate and energy expenditure. This information may be used to make informed decisions about exercise routines and fitness. However, there is limited research that explores the validity and reliability of these devices in non-traditional exercise settings such as pickleball.

As the popularity of pickleball has increased, so has the quantity of scientific research surrounding the activity, with an emphasis on both psychological and physiological responses. Several perceptual investigations have been conducted, attempting to determine motivations for participating in pickleball [7], its association with psychological well-being [8], and the potential to decrease depression [9]. Along with the growth in popularity has come an increase in injury rates [4,10,11]. Consequently, it is necessary to develop a clear understanding of the physiological responses associated with playing pickleball. One doubles pickleball match in individuals averaging 65 years of age induced a moderate physiological response (average HR = 109 bpm, heart rate reserve = 51%, VO_2_ reserve = 53%, kcal per minute = 6) [12]. Another investigation employing doubles pickleball in individuals averaging 38 years of age reported the physiological response to be greater than self-paced walking (peak HR pickleball = 152 bpm, walking = 129 bpm; average HR pickleball = 119 bpm, walking = 105 bpm; total energy expended pickleball = 242 kcal, walking = 179 kcal) [13]. Finally, a comparison between singles and doubles pickleball players (average age between 61 and 63 years old) found similarities between physiological parameters (mean HR singles = 112 bpm, doubles = 112 bpm; percent of maximum HR singles = 70%, doubles = 71%; maximum HR singles = 144 bpm, doubles = 141 bpm) despite doubles players engaging in the activity for a greater period of time (singles = 71 min, doubles = 106 min) [14]. An interesting component of the study conducted by Webber et al. was that heart rate measurements were obtained using wearable technology (Garmin fēnix 5); however, accuracy measures of the devices were obtained and reported in only two individuals [14]. This highlights the need to determine the accuracy of wearables in a variety of use cases, including pickleball.

As previously noted, wearable technology has been a persistent fitness trend over the last five years [5], yet its application in the context of pickleball remains largely unexplored. The existing literature that does incorporate both wearable technology and pickleball only briefly touches on their combined application. A viewpoint piece aimed at clinicians discussed the utilization of fitness trackers and used pickleball as an example to illustrate the limitations of wearable technology regarding the interpretation of movement. It explained how a two-hour game of pickleball would not be interpreted by a wrist-worn device as continuous activity but rather as “bursts of acceleration” [15]. Recently, the concurrent validity of the SwingVision application was reported for performance-based variables during tennis, and it was suggested that the app could be beneficial for pickleball players [16]. A Fitbit HR device was used as a tool to monitor moderate to vigorous physical activity levels in older adults over two weeks of participation in pickleball [8]. Finally, a Garmin fēnix 5 was used to obtain heart rate, activity intensity, and steps during acute pickleball activities. However, the data were not analyzed for validity or reliability (Webber et al. 2023) [14]. While some literature has tangentially associated wearable technology with pickleball, there has not been an appropriately designed study on the validity and reliability of wearables in a pickleball use case to date.

We relish the opportunity to provide users with data related to accuracy and consistency. Thus, the purpose of this study was to evaluate the ability of common wearable technology devices to return valid and reliable data for heart rate and estimated energy expenditure during a simulated singles pickleball activity. It was hypothesized that devices would return valid and reliable heart rate data, but that this would not be the case when estimated energy expenditure was evaluated.

## 2. Materials and Methods

### 2.1. Participants

Twenty adult participants (female n = 7, male n = 12, prefer not to disclose n = 1; age = 44.45 ± 19.6 years; height = 173.0 ± 8.8 cm; mass = 71.35 ± 11.4 kg) were selected for this study after completing a written informed consent form that was approved by the Institutional Review Board (IRB approval #UNLV-2023-242) followed by a health risk questionnaire to determine study eligibility. Participants were recruited for this study via convenience sampling. Our previous work using the same devices to evaluate heart rate validity revealed actual power ranging from 0.8034 to 0.9168 with accompanying sample sizes between 5 and 12 participants [17]. To be conservative, and because we were including the measurement of energy expenditure, we aimed to test 20 participants, which was an early recommendation by the American National Standards Institute/Consumer Technology Association with respect to physical activity monitoring for fitness wearables [18].

### 2.2. Protocol

**Figure 1:**
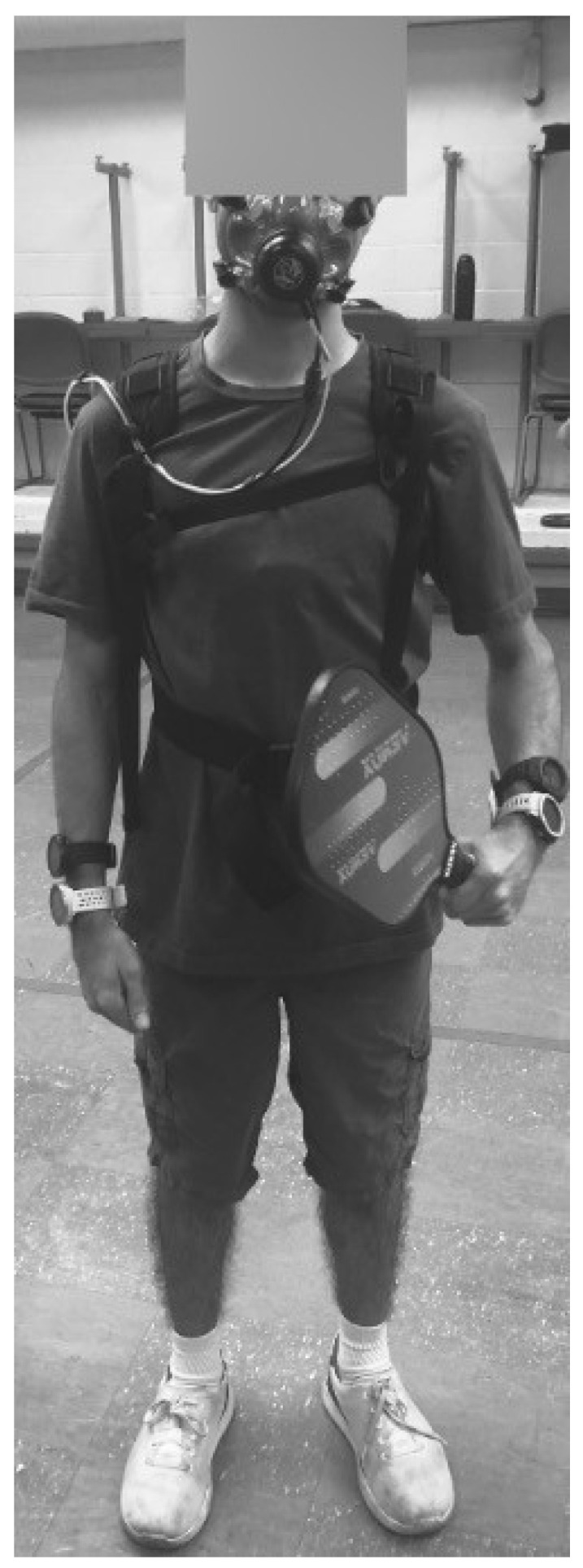
Example of criterion and experimental device setup utilized in the investigation.

The setup for this study was similar to previously completed investigations that allowed us to determine concurrent validity and reliability in the same wearable devices (see Figure 1, [17,19]). Participants were outfitted with a COSMED K5 portable metabolic unit (Rome, Italy) and a Polar H10 chest strap heart rate monitor (Polar Electro Inc., Kempele, Finland), which served as the criterion measures for energy expenditure and heart rate, respectively. The K5 was secured to participants’ backs with small sampling lines connected to a facemask, and the Polar H10 was worn around the chest. The experimental devices used in this investigation were a Garmin Instinct watch (Olathe, KS, USA), Polar Vantage M2 watch (Kempele, Finland), Polar OH1 (Kempele, Finland), and Polar Verity (Kempele, Finland). Two Garmin Instinct watches were worn on the right wrist, and two Polar Vantage M2 watches were worn on the left wrist. A Polar OH1 and Polar Verity were both placed on each bicep. Outcome measures included pulmonary, heart rate, and metabolic measures. Pulmonary measures included ventilation (VE [L/min]), tidal volume (VT [L]), and respiratory frequency (Rf [breaths per min]). Heart rate measures were average and maximum heart rate (HR [beats per minute (bpm)]). Metabolic measures were relative VO_2_ (mL/kg/min), Metabolic Equivalents (METS), Percent of Calories from Fat (FAT%), Percent of Calories from Carbohydrate (CHO%), and Respiratory Quotient (RQ).

This study consisted of a single testing day of acute singles pickleball activity. A simulated match was completed against a member of the research team. Two locations were utilized: (1) an indoor court set up to be of nearly regulation size, and (2) outdoor pickleball courts at Lake Las Vegas Sports Club. Participants played a casual bout of pickleball for a total of 10 min. The total time was split into two five-minute sessions of pickleball with a five-minute rest period in between the sessions. Participants also switched their playing hand for each 5 min session (if a participant used their dominant hand for the first 5 min, they played with their non-dominant for the remaining 5 min). The starting hand (dominant or non-dominant) was counterbalanced.

### 2.3. Data Analysis

The data were collected via three methods: 1. Polar devices were paired to the PerformTek Data Collector application (Valencell, Inc. Raleigh, NC, USA), which collected and combined the data into a single .csv file per trial, or 2. downloaded from the Garmin website as a .tcx file and converted to .csv for each trial, or 3. recorded on the default OMNIA software for the COSMED K5 and exported as an Excel file. The data were compiled using Tableau Prep (Seattle, WA, USA) and viewed in Google Sheets (Mountainview, CA, USA). Statistics were run in Google Sheets, jamovi statistics software (Version 2.3.28, Sydney, Australia), IBM SPSS statistics software (Version 28.0.1.0, Armonk, NY, USA), or an r Shiny app (Shinyapps.io by Posit). Descriptives, error analysis, linearity assessment, equivalence testing, and Bland–Altman plots were performed in Google sheets or jamovi, while cross-correlations were performed in SPSS and repeated measures correlation (RM-correlation) was performed via a Shiny App (https://lmarusich.shinyapps.io/shiny_rmcorr/ URL accessed on 26 March 2024). Conversion of .tcx to .csv files was performed using custom python code run within the OpenAI python environment using the Data Analyst GPT from ChatGPT 4 in March 2024. Error analysis was tested via mean absolute percentage error (MAPE) in Google Sheets. Linearity was assessed via multiple correlation coefficients, including Pearson’s Product Moment Correlation Coefficient, Lin’s Concordance Correlation Coefficient, and RM-Correlation, as well as Deming Regression. Equivalence testing was performed utilizing the confidence interval for difference in means method with upper and lower thresholds being set at ±10% of the criterion mean. Bland–Altman plots were generated with mean bias and 95% confidence intervals and limits of agreement also being reported. Cross-correlations were performed to ensure there was not a lag in the data compared to the criterion. Measures associated with reliability include the coefficient of variation (CV) and a two-way mixed model with an absolute agreement intraclass correlation coefficient (ICC). The CV was determined using Excel (Microsoft Excel for Mac version 16.66.1, Redmond, WA, USA) and the ICC (single measures) using SPSS Statistics (IBM SPSS Statistics, version 28.0.1.0, Armonk, NY, USA). Predetermined thresholds of ≤10% for MAPE, ≥0.70 for linearity measures, ≤10% for CV, and ≥0.70 for ICC, with a lower bound of the 95% CI ≥ 0.70 were used.

## 3. Results

**Figure 2:**
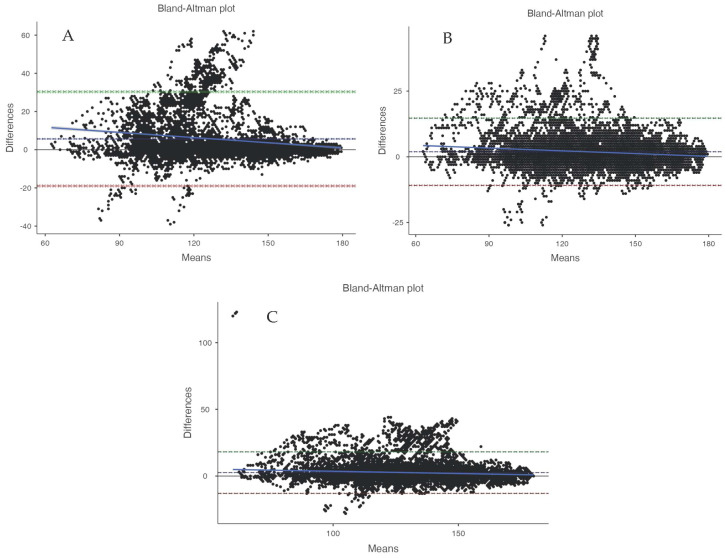
Representative Bland–Altman plots denoting visual agreement of the heart rate (bpm) measured in wearable technology devices ((**A**) = Garmin Instinct, (**B**) = Polar Verity Sense, (**C**) = Polar OH1) and a criterion measure (Polar H10) in participants (*N* = 20) who completed 10 min of simulated pickleball gameplay. The colored bands represent the 95% CI for upper and lower limits of agreement and the bias.

**Figure 3:**
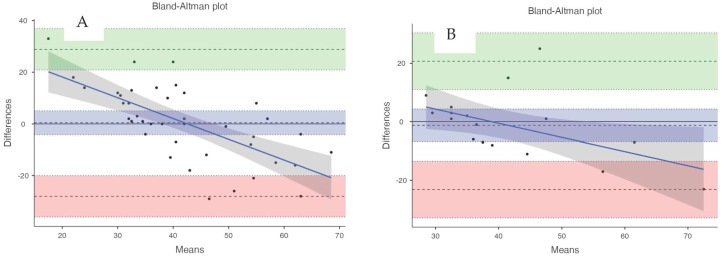
Representative Bland–Altman plots denoting visual agreement of accumulated estimated energy expenditure (kcal) measured in wearable technology devices ((**A**) = Garmin Instinct, (**B**) = Polar Vantage M2) and a criterion measure (COSMED K5) in participants (*N* = 20) who completed 10 min of simulated pickleball gameplay. The colored bands represent the 95% CI for upper and lower limits of agreement and the bias.

Of the 20 participants in this study during pickleball gameplay, an average heart rate of 130.89 ± 21.02 bpm from the Polar H10 and an average energy expenditure of 42.28 ± 9.03 kcal from the COSMED K5 were observed, respectively (criterion measures for heart rate and energy expenditure). Heart rate error analysis displayed a MAPE of ≤10% (see Table 1) and ≥10% for energy expenditure data (see Table 2). Linearity was determined through correlation analysis in all devices, yielding ≥ 0.7 in heart rate data (Table 1) and <0.7 in energy expenditure data (Table 1). Equivalence testing results were supported in the heart rate data (see Table 1) but not in the energy expenditure data (see Table 2). Bland–Altman bias was used to assess the agreement of heart rate and energy expenditure data, with 95% confidence intervals (see Table 1 and Table 2, and Figure 2 and Figure 3). We are unable to conclude that agreement occurred in either the heart rate or estimated energy expenditure in any of the devices (see Table 1 and Table 2, and Figure 2 and Figure 3).

### Sex Disaggregated Data

Because the Sex and Gender Equity in Research (SAGER) guidelines encourage the reporting of disaggregated data [20], we are presenting metabolic variables derived from the criterion device below (see Table 3). Since the primary aim of the study was not directed toward determining whether sex differences exist, we did not test for any sex or gender differences, nor is the study powered for such a comparison. We provide the data below to align with SAGER guidelines and to assist with future meta-analyses.

## 4. Discussion

The purpose of this study was to evaluate the ability of wearable technology devices to return valid and reliable data for heart rate and estimated energy expenditure during a simulated singles pickleball activity. It was hypothesized that devices would return valid and reliable heart rate data but not estimated energy expenditure. Our hypothesis was partially correct, as devices were valid and reliable when returning heart rate measurements, but the devices fell short when considering estimated energy expenditure. No device met the threshold for acceptable agreement for either variable.

Our findings reveal sufficient evidence to conclude that all four devices (Garmin Instinct, Polar Vantage M2, Polar OH1, and Polar Verity Sense) were reliable and valid when considering heart rate measurements obtained during simulated singles pickleball gameplay. This conclusion aligns with previous literature. The Polar OH1 has been validated against accepted Polar chest strap devices (H7, H10), and was reported to have acceptable agreement with measurements in all intensity ranges and during different activities, such as sprinting [21], swimming [22], and power vinyasa yoga [23]. It is also important to note that the Polar OH1 has been shown to correlate well with ECG measurements; however, it was found to be more accurate at higher levels of intensity due to increased blood flow to the extremities [24]. The Polar OH1 has been shown to be reliable and valid for endurance sports, though excessive arm swing can lower the reliability of measurements [25]. The Polar Verity Sense was found reliable and valid in trail running [17] and in walking [26]. However, during activities where more arm movements are required, it is found to be less reliable [27]. The Polar Vantage M2 was found to be reliable during lower intensity activities but became less so as intensity increased [28]. Lastly, preliminary evidence suggests the Garmin Instinct may be reliable but not valid for maximum and average heart rate [29,30]. Similar findings suggest that the Polar Vantage M2 may not return valid heart rates during resistance training [31].

When considering the evaluation of energy expenditure, much less literature exists for validity and reliability measures than heart rate. Our laboratory group reported that accumulated calories estimated from both the Garmin Instinct and the Polar Vantage M2 performed poorly across all measures of validity during self-paced walking, jogging, and skipping [19]. While the Garmin Instinct did not meet our predetermined thresholds for reliability in any activity, the Polar Vantage M2 was considered reliable during walking and jogging but not skipping [19]. Additionally, a recent conference abstract reported the Garmin Instinct met neither the threshold for validity nor reliability in participants who completed circuit resistance training exercises engaging both the upper (push-ups and shoulder press) and lower body (front squat and reverse lunge) [32]. The return of energy expenditure in commercial wearables is notably poor [33], and aligns with reported findings for the Garmin vívosmart HR+ [34], the Garmin vívofit [35], and the Polar Vantage V [36]. We add our findings to the accumulating evidence that consumer wearable devices return poor estimates of energy expenditure. In this case, we can specifically state that the Garmin Instinct returns energy estimates that are neither valid nor reliable, while the Polar Vantage M2 returns estimates that are reliable but not valid. As we have noted previously, having consistent measures are meaningless if validity assumptions are violated [19].

The primary real-world application of this research is that the wearable devices analyzed can help individuals track the intensity of their pickleball sessions by providing appropriate heart rate data. Many individuals engage in pickleball to improve their cardiovascular endurance [1], and they may choose a target heart rate range to ensure they are training at a suitable intensity level. Thus, it is important that the wearable devices used during pickleball provide accurate and consistent heart rate measurements. Without high-quality measurements, individuals may train at insufficient or excessive intensities, hindering their fitness progress. This research has demonstrated that the Polar OH1, Polar Vantage M2, Polar Verity, and Garmin Instinct devices produce valid and reliable heart rate data during pickleball, allowing individuals to confidently rely on these devices to meet their exercise intensity goals for this sport. Another implication of this research is that individuals using these devices may not be consuming an optimal number of calories to match the energy costs of the sport due to inaccurate and imprecise energy expenditure estimates. This may be problematic if one is engaging in pickleball to manage body weight, as valid and reliable measurements of energy expenditure are important to ensure one’s energy intake is appropriate. Wearable technology manufacturers should focus on improving the energy expenditure measurement capabilities of their devices to provide clients with the highest-level fitness tracking.

A limitation of wearable technology is the amount of missing data, which was a serious concern for several devices. For HR analysis, the Garmin wrist-worn devices were missing up to 8900 s of data compared to the criterion, which represents 62.8% missing data. Additionally, for the energy expenditure measurements, one of the Polar Vantage M2 units had only 10 of 40 trials with results, though this was due to a technical error in syncing and not device error, but it should still be noted that there were large amounts of missing data for the energy expenditure analysis. The missing data points associated with the experimental devices likely affected agreement as interpreted through the Bland–Altman analysis. Because of this, it is possible that Bland–Altman assumptions were not met (measurement methods have the same precision; the precision is constant) [37]. While the Bland–Altman analysis is included in the majority but not all wearable research, future investigations should evaluate its utility, as our findings did not align with other validity measures in the current investigation. Relatedly, although not a strict limitation, there is an important concern when measuring the validity and reliability of wearable technology during pickleball play. Generally, devices were valid and reliable (but not in agreement) when the heart rate measurement was considered but not with respect to caloric return. Such an incongruence is strange, given that caloric expenditure algorithms purportedly rely on heart rate to estimate calories expended. Devices that are accurate across all returned estimates or measurements are needed. Finally, as this study was not designed to compare sex or gender differences, future studies may concentrate on heart rate and energy expenditure in wearable technology within and among these groups.

## 5. Conclusions

In conclusion, pickleball, as a fast-growing sport, requires research into the performance of wearable devices for monitoring heart rate and energy expenditure. In the context of current research, the Garmin Instinct, Polar OH1, Polar Verity, and Polar Vantage M2 met all thresholds for heart rate measurement to be considered both valid and reliable during pickleball. The Garmin Instinct was neither valid nor reliable in returning estimated energy expenditure during pickleball, while the Polar Vantage M2 was reliable but not valid. From a broader perspective, algorithms for wearable devices to estimate energy expenditure need to be refined until they achieve the same level of accuracy currently seen for heart rate, specifically, improvements in detecting diverse movement patterns. Given pickleball’s unique physical demands and its popularity among varied age groups, it may be a great exercise to consistently test these measures, as wearable activity devices continue to develop and evolve.

## Figures and Tables

**Table 1:** Heart rate validity and reliability during simulated singles pickleball gameplay.

	Polar H10	Garmin Instinct 1	Garmin Instinct 2	Polar Verity Sense 1	Polar Verity Sense 2	Polar OH1 1	Polar OH1 2
Average (bpm)	130.89	124.78	124.42	129.32	129.19	128.32	130.59
Standard Deviation	21.02	23.93	24.41	22.04	21.91	21.97	21.52
Count	14,291	6163	5310	13,682	14,191	13,853	10,474
Variance	441.97	572.54	595.87	485.73	479.85	482.77	463.18
MAPE		6.32%	5.73%	2.97%	2.92%	3.39%	3.45%
Pearson Correlation		0.85	0.88	0.96	0.96	0.93	0.94
Lin’s Concordance		0.82	0.84	0.95	0.96	0.93	0.94
Repeated Measures Correlation		0.87	0.89	0.92	0.94	0.92	0.90
Mean Difference		5.67	5.34	1.91	1.84	2.58	2.25
SE Difference		0.16	0.16	0.06	0.05	0.07	0.07
Lower 90% CI Difference		5.41	5.09	1.82	1.76	2.47	2.13
Upper 90% CI Difference		5.94	5.60	2.01	1.92	2.70	2.37
Equivalence Interval (10% of criterion average)	13.09						
Equivalence Testing Result		Equivalence Supported	Equivalence Supported	Equivalence Supported	Equivalence Supported	Equivalence Supported	Equivalence Supported
Deming Regression Slope		−19.06	−24.18	−6.69	−7.59	−7.19	−2.90
Deming Regression Intercept		1.10	1.15	1.04	1.04	1.04	1.00
Bland–Altman Bias		5.67 (5.36, 5.99)	5.34 (5.04, 5.65)	1.91 (1.80, 2.02)	1.87 (1.74, 1.94)	2.58 (2.45, 2.72)	2.25 (2.11, 2.39)
Bland–Altman Lower Limit of Agreement		−18.98	−17.06	−10.87	−9.97	−12.92	−12.20
Bland–Altman Upper Limit of Agreement		30.32	27.75	14.70	13.65	18.09	16.69
Bland–Altman *t*-statistic, *p*-value		35.33, <0.001	33.99, <0.001	34.18, <0.001	36.26, <0.001	38.31, <0.001	31.04, <0.001
Bland–Altman regression, *p*-value		0.065, <0.001	0.167, <0.001	0.065, <0.001	0.103, <0.001	0.029, <0.001	0.044, <0.001
Reliability Testing							
CV		9.30%		2.68%		5.01%	
SEM (bpm)		11.5861		3.4655		6.4797	
ICC (two-way fixed)		0.77 (0.76, 0.78)		0.98 (0.97, 0.98)		0.91 (0.91, 0.91)	

MAPE = Mean Absolute Percentage Error, SE = Standard Error, CI = Confidence Interval, CV = coefficient of variation, SEM = standard error of the mean, ICC = Intraclass Correlation.

**Table 2:** Energy expenditure validity and reliability during simulated singles pickleball gameplay.

	COSMED K5	Garmin Instinct 1	Garmin Instinct 2	Polar Vantage M2 1	Polar Vantage M2 2
Accumulated total (kcal)	42.28	41.85	49.33	42.06	39.40
Standard Deviation	9.03	17.84	17.92	15.40	19.48
Count	40	40	40	18	10
Variance	81.54	318.34	321.10	237.23	379.60
MAPE		28.08%	27.67%	18.87%	23.38%
Pearson Correlation		0.59	0.69	0.69	0.71
Lin’s Concordance		0.47	0.49	0.63	0.62
Mean Difference		0.42	−7.05	−1.22	3.9
SE Difference		2.3	2.12	2.64	4.36
Lower 90% CI Difference		−3.44	−10.62	−5.81	−4.09
Upper 90% CI Difference		4.29	−3.48	3.36	11.89
Equivalence Interval (10% of criterion average)	4.2275				
Equivalence Testing Result		Equivalence Not Supported	Equivalence Not Supported	Equivalence Not Supported	Equivalence Not Supported
Deming Regression Slope		2.85	2.55	1.8	1.92
Deming Regression Intercept		−78.83	−58.28	−31.46	−43.94
Bland–Altman Bias		0.42 (−4.22, 5.07)	−7.05 (−11.34, −2.76)	−1.22 (−6.78, 4.34)	3.90 (−5.96, 13.76)
Bland–Altman Lower Limit of Agreement		−28.03	−33.35	−23.15	−23.11
Bland–Altman Upper Limit of Agreement		28.88	19.25	20.7	30.91
Bland–Altman *t*-statistic, *p*-value		0.19, 0.854	3.32, 0.002	0.46, 0.649	0.89, 0.394
Bland–Altman regression, *p*-value		0.538, <0.001	0.607, <0.001	0.377, <0.001	0.377, 0.123
Reliability Analysis					
CV		14.69%		6.01%	
SEM (kcal)		6.6948		2.4725	
ICC (two-way fixed)		0.86		0.98	

MAPE = Mean Absolute Percentage Error, SE = Standard Error, CI = Confidence Interval, CV = coefficient of variation, SEM = standard error of the mean, ICC = Intraclass Correlation.

**Table 3:** Sex disaggregated metabolic data during simulated singles pickleball gameplay.

Sex	Hand	RF (br/min)	VT (L)	VE (L/min)	RER	VO_2_ (mL/Kg/min)	FAT%	CHO%
Female (n = 7)	Dominant	36.44 ± 5.44	1.09 ± 0.13	39.74 ± 8.88	0.79 ± 0.07	21.00 ± 4.17	70.90 ± 22.51	29.10 ± 22.51
	Non	37.90 ± 4.76	1.06 ± 0.15	39.60 ± 5.01	0.79 ± 0.06	20.70 ± 2.31	69.57 ± 19.39	30.43 ± 19.39
Male (n = 13)	Dominant	38.74 ± 6.06	1.49 ± 0.34	57.31 ± 13.20	0.82 ± 0.08	27.12 ± 5.52	59.48 ± 24.21	40.52 ± 24.21
	Non	39.94 ± 5.43	1.38 ± 0.27	54.26 ± 11.28	0.83 ± 0.05	24.68 ± 4.27	58.31 ± 17.63	41.69 ± 17.63

Note: RF = respiratory frequency, VT = tidal volume, VE = minute ventilation, RER = respiratory exchange ratio, VO_2_ = oxygen consumption.

## Data Availability

The data presented in this study are available on request from the corresponding author.
